# Novel (1*E*,3*E*,5*E*)-1,6-*bis*(Substituted phenyl)hexa-1,3,5-triene Analogs Inhibit Melanogenesis in B16F10 Cells and Zebrafish

**DOI:** 10.3390/ijms19041067

**Published:** 2018-04-03

**Authors:** Jisun Oh, Jungeun Kim, Jin Ho Jang, Sangwoo Lee, Chul Min Park, Woo-Keun Kim, Jong-Sang Kim

**Affiliations:** 1 School of Food Science and Biotechnology (BK21 Plus), Kyungpook National University, Daegu 41566, Korea; j.oh@knu.ac.kr (J.O.); astely88@hanmail.net (J.K.); mk5266@naver.com (J.H.J.); 2 System Toxicology Research Center, Korea Institute of Toxicology, Daejeon 34114, Korea; sangwoo.lee@kitox.re.kr; 3 Center for Convergent Research of Emerging Virus Infection, Korea Research Institute of Chemical Technology, Daejeon 34114, Korea; parkcm@krict.re.kr

**Keywords:** synthetic compound, anti-melanogenesis, melanocyte, zebrafish, pigmentation

## Abstract

The present study aimed to evaluate the anti-melanogenic activity of 1,6-diphenyl-1,3,5-hexatriene and its derivatives in B16F10 murine melanoma cells and zebrafish embryos. Twenty five (1*E*,3*E*,5*E*)-1,6-*bis*(substituted phenyl)hexa-1,3,5-triene analogs were synthesized and their non-cytotoxic effects were predictively analyzed using three-dimensional quantitative structure-activity relationship approach. Inhibitory activities of these synthetic compounds against melanin synthesis were determined by evaluating melanin content and melanogenic regulatory enzyme expression in B16F10 cells. The anti-melanogenic activity was verified by observing body pigmentation in zebrafishes treated with these compounds. Compound **#2**, **#4**, and **#6** effectively decreased melanogenesis induced by α-melanocyte-stimulating hormone. In particular, compound **#2** remarkably lowered the mRNA and protein expression levels of microphthalmia-associated transcription factor (MITF), tyrosinase (TYR), tyrosinase-related protein 1 (TYRP1), and TYRP2 in B16F10 cells and substantially reduced skin pigmentation in the developed larvae of zebrafish. These findings suggest that compound **#2** may be used as an anti-melanogenic agent for cosmetic purpose.

## 1. Introduction

Melanin is a dark pigment produced through melanogenesis, a natural phenomenon occurring by the action of tyrosinase in melanocytes [1]. Tyrosinase converts tyrosine to l-3,4-dihydroxyphenylalanine (l-DOPA), dopaquinone, dopachrome, and subsequently to melanin. Upon the exposure of the skin to external stimuli such as ultraviolet light, air pollution, and oxidative stress, melanin pigments are excessively produced within melanocytes. The pigments are then transferred to the keratinocytes and, thus, accumulate in the epidermal layer of the skin, which consequently causes skin darkening and cancer [2,3,4]. Therefore, the prevention of melanin synthesis in melanocytes for the improvement of skin health is commonly studied based on the following three criteria: (i) development of tyrosinase inhibitors; (ii) development of bioactive chemicals that are toxic to melanocytes; and (iii) development of DOPA-reducing substances to prevent the oxidation of intermediate metabolites in the melanogenic pathway [5,6].

Given that tyrosinase is the key enzyme in melanin synthesis that exclusively occurs in melanocytes, melanogenesis may be effectively inhibited by antagonizing the catalytic activity and/or biosynthesis of tyrosinase [5,6,7,8,9]. Most commercially available skin-whitening agents are tyrosinase inhibitors [9,10,11] such as kojic acid [12], arbutin [13], hydroquinone [14], ellagic acid [15], and their derivatives. All of these have drawbacks, including carcinogenicity, chemical instability, and poor bioavailability [9,10].

Ha and coworkers have recently synthesized a variety of triene analogs as potential tyrosinase inhibitors and examined the structure-activity relationship between mushroom tyrosinase and these synthesized compounds [16]. These authors found that certain triene derivatives, including 4,4′-((1*E*,3*E*,5*E*)-hexa-1,3,5-triene-1,6-diyl)diphenol, 5,5′-((1*E*,3*E*,5*E*)-hexa-1,3,5-triene-1,6-diyl)bis(2-methoxy-phenol), and 5,5′-((1*E*,3*E*,5*E*)-hexa-1,3,5-triene-1,6-diyl)dibenzene-1,3-diol, may possibly act as pharmacophores in the process of tyrosinase inhibition. In the present study, we evaluated the potential anti-melanogenic effect of novel synthetic triene analogs—1,6-diphenyl-1,3,5-hexatriene and its derivatives, using B16F10 murine melanoma cells and zebrafish embryos.

## 2. Results

### 2.1. Synthesis of Novel Compounds

A total of 25 compounds were synthesized [16] and their chemical identifies are presented in Supplementary Figure S1 and Supplementary Table S1. Results of the in silico analysis using QSAR method showed that all the compounds had no predicted toxicity, as observed from the test for sensitization and skin irritation.

### 2.2. Cytotoxicity of the Compounds

**Figure 1:**
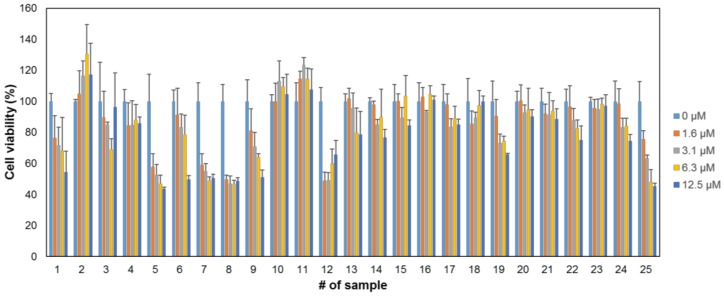
Cytotoxicity of novel synthetic compounds. B16F10 murine melanoma cells were treated with each compound at various concentrations (0, 1.6, 3.1, 6.3, and 12.5 μM) for 24 h. CCK-8 assay was performed to assess the cytotoxic effect. *n* = 3; error bars, mean ± SD.

The compounds were examined for their cytotoxicities. Murine melanoma B16F10 cells were treated with each compound at various concentrations (0, 1.6, 3.1, 6.25, and 12.5 μM). After 24 h of incubation, the cell viability was assayed using CCK-8 kit (Figure 1). The majority of compounds, including **#2**, **#3**, **#4**, **#6**, **#10**, **#11**, **#13**, **#14**, **#15**, **#16**, **#17**, **#18**, **#20**, **#21**, **#22**, **#23**, and **#24**, showed negligible toxicity at the maximum concentration of 12.5 μM. Eight compounds (**#1**, **#5**, **#7**, **#8**, **#9**, **#12**, **#19**, and **#25**) were found to be cytotoxic. The compounds with little cytotoxicity were further analyzed for melanogenesis inhibition activity.

### 2.3. In Vitro Anti-Melanogenic Effect of the Compounds

**Figure 2:**
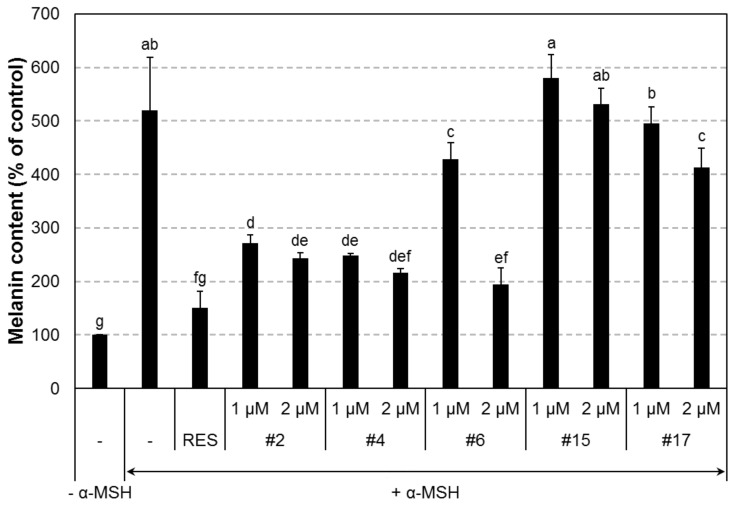
In vitro anti-melanogenic activity of compound **#2**, **#4**, **#6**, **#15**, and **#17**. B16F10 cells were cultured in the presence of various compounds at 1 or 2 μM concentration and/or α-MSH at 10 nM for 72 h. Cellular melanin content decreased following treatment with these compounds in a dose-dependent manner. α-MSH, alpha-melanocyte-stimulating hormone (10 nM). RES, resveratrol (10 μM). *n* = 3; error bars, mean ± SEM. Different alphabetical letters indicate significant differences among the conditions (*p* < 0.05).

**Figure 3:**
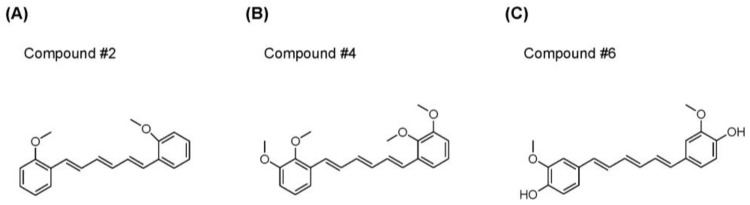
Chemical structure of anti-melanogenic compounds **#2** (**A**), **#4** (**B**), and **#6** (**C**).

To investigate the melanogenesis inhibition effect of the non-cytotoxic synthetic compounds (Supplementary Figure S2), B16F10 cells were stimulated with α-MSH (10 nM) and simultaneously treated with each compound at either 1 or 5 μM concentration. After 72 h of treatment, relative melanin content produced in the cells was spectrophotometrically measured. Among the compounds, **#2**, **#4**, **#6**, **#15**, and **#17** decreased the melanin content and were chosen for verification by testing at either 1 or 2 μM. The compounds, **#2**, **#4**, **#6**, and **#17** were found to inhibit α-MSH-induced melanin production in a concentration-dependent manner (Figure 2). In particular, compound **#2**, **#4**, and **#6** significantly inhibited melanogenesis in B16F10 cells at 1 μM concentration in the presence of α-MSH. The chemical structure of each of the three compounds is shown in Figure 3. These compounds were used for further examinations.

### 2.4. Tyrosinase Inhibition Activity of the Selected Compounds

**Figure 4:**
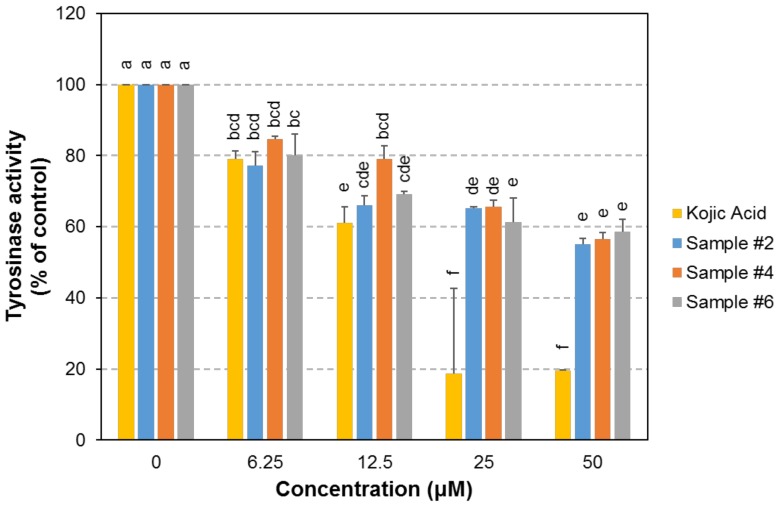
Tyrosinase inhibitory effect of compound **#2**, **#4**, and **#6**. Catalytic activity of the mushroom tyrosinase was measured in the presence of each compound at various concentrations (0, 6.25, 12.5, 25, and 50 μM). Kojic acid, a well-known tyrosinase inhibitor, was used as a positive control. *n* = 3; error bars, mean ± SEM. Different alphabetical letters indicate significant differences among the conditions (*p* < 0.05).

The three selected compounds, **#2**, **#4**, and **#6**, were further examined for tyrosinase inhibition activity using mushroom tyrosinase (Figure 4) or using crude tyrosinase extract from B16F10 cells (Supplementary Figure S3). Kojic acid, a well-known tyrosinase inhibitor, was used as a positive control. The result demonstrated that the compounds **#2** and **#4** effectively inhibited the catalytic activity of tyrosinase and their inhibitory effects were comparable to the effect of kojic acid at concentrations of ≤12.5 μM.

### 2.5. Inhibition of the Transcript and Protein Expression of MITF and Melanogenic Enzymes

**Figure 5:**
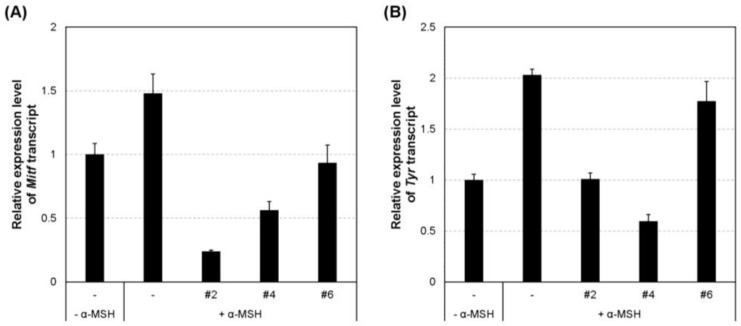

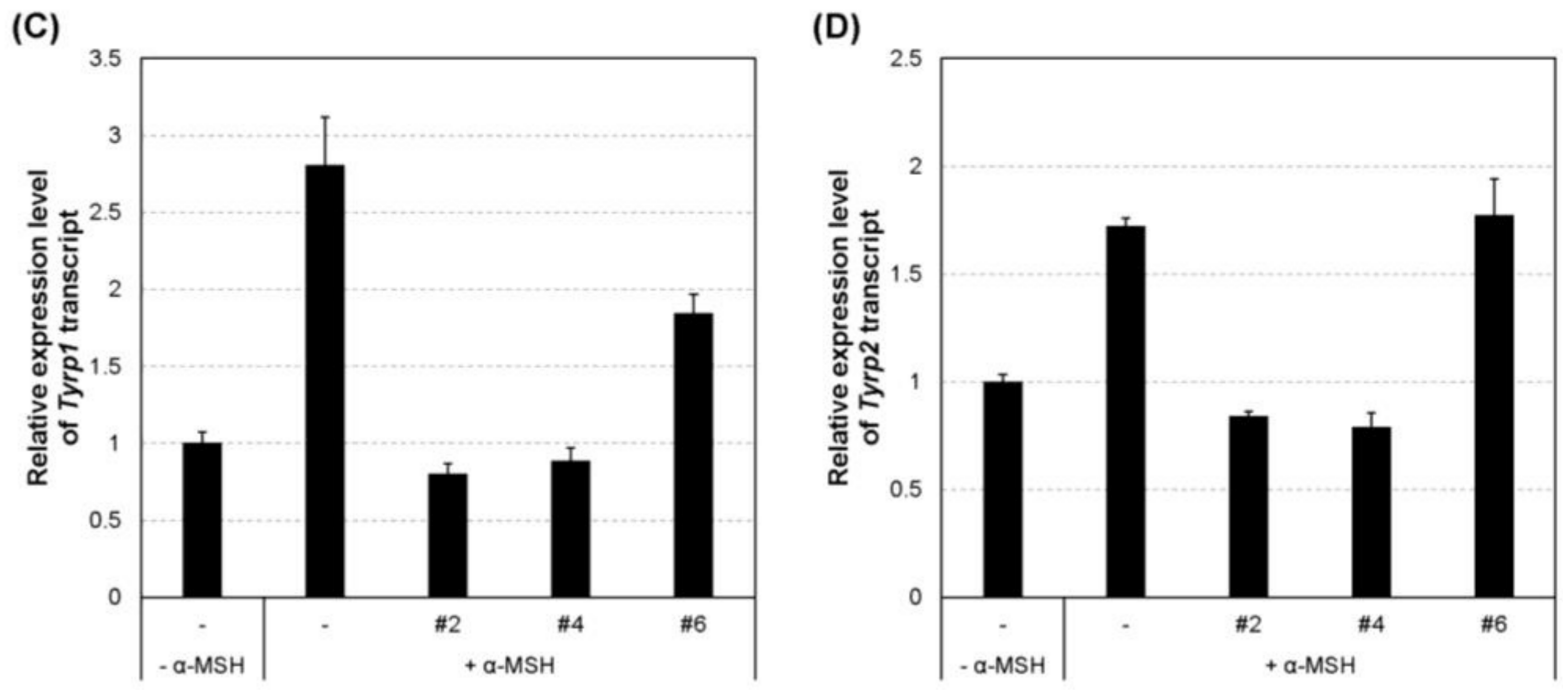
Relative mRNA transcript levels of melanogenic genes *Mitf*, *Tyr*, *Tyrp1*, and *Tryp2*. (**A**) *Mitf* mRNA level; (**B**) *Tyr* mRNA level; (**C**) *Tyrp1* mRNA level; (**D**) *Tyrp2* mRNA level. B16F10 cells were treated with one of three compounds (**#2**, **#4**, and **#6**) at 2 μM for 24 h. The relative mRNA levels for indicated genes in the cells were quantified using SYBR green-based real-time PCR and normalized to the *β-actin* mRNA level. The information of primer sets is shown in Table 1. α-MSH, α-melanocyte-stimulating hormone; *Mitf*, microphthalmia-associated transcription factor; *Tyr*, tyrosinase; *Tyrp1*, tyrosinase-related protein 1; *Tryp2*, tyrosinase-related protein 2.

The effects of the selected compounds on the expression of mRNA encoding melanogenic factors were evaluated in α-MSH-stimulated B16F10 cells. Total mRNAs were extracted from the cells treated with α-MSH in the absence or presence of each compound. The mRNA levels of tyrosinase (*Tyr*), tyrosinase-related protein 1 (*Tyrp1*), *Tryp2*, and microphthalmia-associated transcription factor (*Mitf*) were quantified by real-time PCR (Figure 5). The mRNA levels of *Tyr*, *Tyrp1*, *Tryp2*, and *Mitf* induced by α-MSH were effectively suppressed in the presence of compounds **#2** and **#4**.

**Figure 6:**
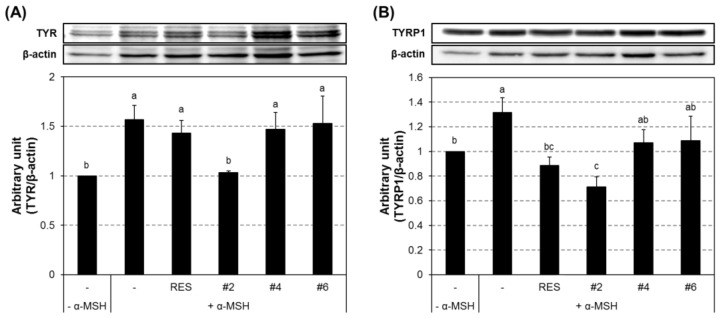

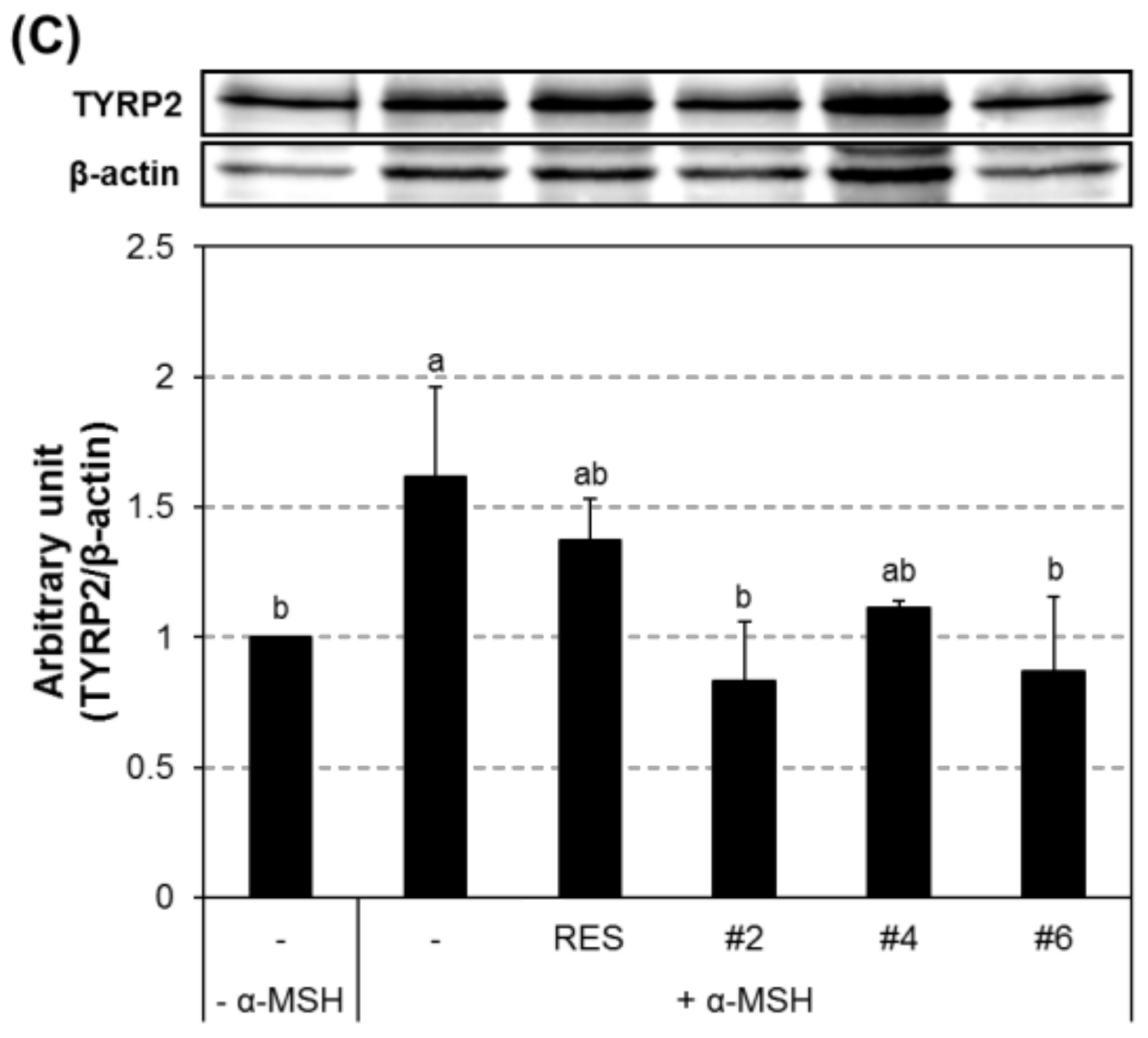
Melanogenic protein expression in the compound-treated B16F10 cells. The cells were treated with the indicated compound at 2 μM concentration for 24 h. Relative protein expression levels of cytoplasmic TYR (**A**), TYRP1 (**B**), and TYRP2 (**C**) were determined by western blot analysis. α-MSH, alpha-melanocyte-stimulating hormone (10 nM); RES, resveratrol (10 μM); TYR, tyrosinase; TYRP1, tyrosinase-related protein 1; TRYP2, tyrosinase-related protein 2. *n* = 3; error bars, mean ± SEM. Different alphabetical letters indicate significant differences among the conditions (*p* < 0.05).

The expression level of melanogenic proteins in B16F10 cells was examined by Western blot analysis (Figure 6). The intracellular levels of TYR, TYRP1, and TRYP2 were markedly increased in α-MSH-treated cells and greatly reduced by concomitant treatment with compound **#2**. However, compounds **#4** and **#6** were ineffective in suppressing α-MSH-induced expression of TYR and TYRP1; however, compound **#6** induced a prominent inhibition of TYRP2 expression.

### 2.6. Depigmentation in Zebrafish Exposed to Compound ***#2***

**Figure 7:**
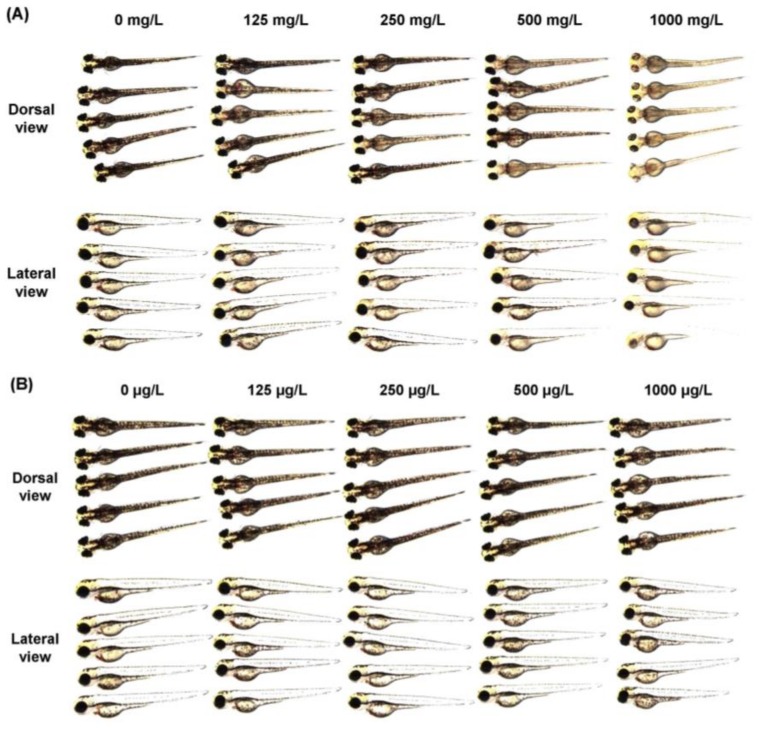

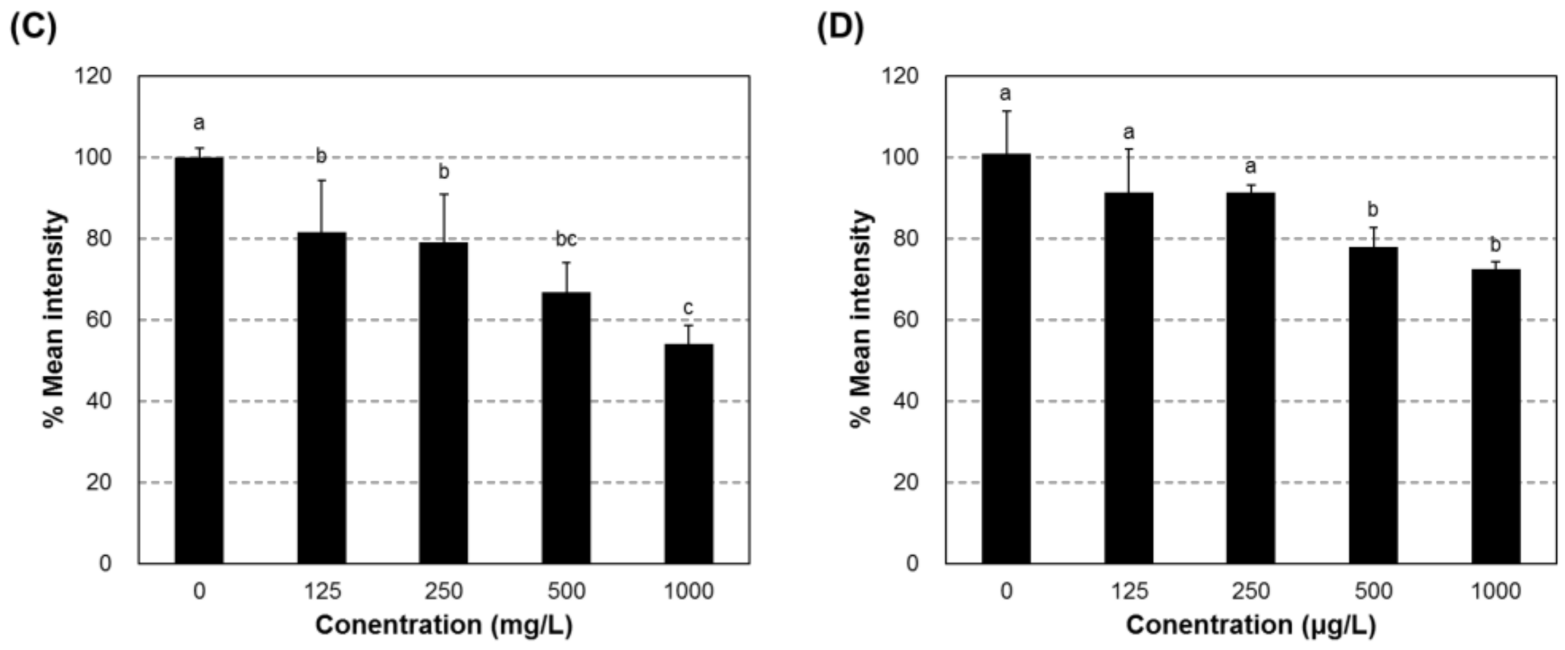
Pigment development in zebrafishes treated with compound **#2**. Zebrafish embryos were exposed to either arbutin (**A**) or compound **#2** (**B**) at indicated doses for 72 h. Upper, dorsal view; lower, lateral view. Pigmentation in zebrafish after exposure to arbutin (**C**) or compound **#2** (**D**) was quantitatively measured. *n* = 5; error bars, mean ± SD. Different alphabetical letters indicate significant differences among the conditions (*p* < 0.05).

The anti-melanogenic activity of compound **#2**, which was most effective in B16F10 cells, was tested in the zebrafish model. Zebrafishes were exposed to either arbutin or compound **#2** at concentrations ranging from 0 to 1000 μg/L for 72 h and the pigmentation on the skin was stereomicroscopically observed (Figure 7A,B). The skin exposed to compound **#2** showed significant depigmentation at 500 and 1000 μg/L (Figure 7C,D). The exposure to compound **#2** at the tested doses failed to affect the viability or activity of zebrafish.

## 3. Discussion

The present study examined the anti-melanogenic potential of novel synthetic compounds for cosmetic purpose. Based on the previous in silico study by the docking simulation implicating triene analogs as pharmacophores inhibiting tyrosinase [16], we hypothesized that 1,6-diphenyl-1,3,5-hexatriene and its derivatives may impede the process of melanogenesis. The hypothesis was examined in the following three ways. First, a total of 25 triene analogs were screened for their cytotoxic effects on melanocytes and inhibitory effect on melanogenesis. Second, the compounds with low cytotoxicity and high anti-melanogenesis activity were selected and evaluated for their abilities to suppress the expression of melanogenic factors in B16F10 murine melanoma cells. The third, compound (**#2**) that exhibited the most effective anti-melanogenesis activity was further examined for its in vivo anti-melanogenic effect in zebrafish.

Multiple reports have demonstrated that tyrosinase may be catalytically inhibited by aromatic acids such as benzoic acid and cinnamic acid as well as their derivatives [11,17,18,19]. Furthermore, 1,6-diphenyl-1,3,5-hexatriene and its derivatives are likely to efficiently penetrate into the lipid bilayer in skin because of their hydrophobicity [20]. In addition, Ha and colleagues reported that synthetic compounds containing triene analogs have stronger tyrosinase inhibitory effect than compounds containing benzene analogs [16]. Therefore, compounds **#2**, **#4**, and **#6**, which are 1,6-diphenyl-1,3,5-hexatriene derivatives, may easily cross the lipid bilayer of skin cells, dock to tyrosinase in the melanocytes, and inhibit its activity. However, the mechanism through which these synthetic molecules are metabolized in the melanocytes remains to be further unraveled.

As expected from QSAR analysis, most of the synthetic compounds tested in this study were non-toxic to B16F10 mouse melanoma cells. In particular, compounds **#2**, **#4**, and **#6** reduced α-MSH-induced melanin synthesis in a dose-dependent manner and their inhibitory effects on the activity of mushroom tyrosinase were comparable with the effect of kojic acid at concentrations of ≤12.5 μM. Among the three compounds, compound **#2** was the most effective and abrogated α-MSH–induced increase in mRNA and protein expression of melanogenic factors such as MITF, TYR, TYRP1, and TYRP2 in B16F10 cells. It is well-known that TYR is modulated by MITF, a master transcription factor in melanogenesis, and that TYRP1 and TYRP2 are major targets of MITF-mediated melanogenic enzymes [21,22,23]. During melanin synthesis, tyrosine undergoes TYR-dependent conversion and is catalyzed to dopaquinone and subsequently dopachrome. TYRP2 converts dopachrome to 5,6-dihydroxyindole-2-carboxylic acid (DHICA), whereas TYRP1 oxidizes DHICA to a carboxylated indole-quinone, which is eventually converted into melanin [24]. Thus, our results suggest that compound **#2**, which suppressed MITF-mediated melanogenic enzyme expression, has a great potential as an anti-melanogenic agent.

The anti-melanogenic effect of compound **#2** was further verified in vivo using zebrafish embryos. Zebrafish are useful as a vertebrate model system to investigate the depigmentation activity of melanogenic regulatory compounds [25]. In this study, zebrafish embryos were treated with compound **#2** at various doses for 72 h. The significant reduction in body pigmentation was observed in a dose-dependent manner without oral toxicity during the experimental period. Although this observation supports the anticipated anti-melanogenic effect of compound **#2** on human melanocytes, further study is essential to examine whether the compound is affordable to manufacture for commercial purpose.

Taken together, we have evaluated the inhibitory activity of a variety of (1*E*,3*E*,5*E*)-1,6-*bis*(substituted phenyl)hexa-1,3,5-triene analogs on melanin synthesis in B16F10 cells and zebrafish embryos. Among the three 1,6-diphenyl-1,3,5-hexatriene derivatives that effectively decreased α-MSH-induced melanogenesis, compound **#2** was found to suppress the mRNA and protein expression of melanogenic enzymes in vitro and in vivo. These findings suggest that this compound may be used as a potent anti-melanogenic agent for cosmetic purposes.

## 4. Materials and Methods

### 4.1. Preparation of Synthetic Compounds

A total of 25 synthetic compounds, 1,6-diphenyl-1,3,5-hexatriene and its derivatives, were synthesized, as previously described (Supplementary Figure S1) [16] with minor modifications. The synthetic details were described in Supplementary Table S1. The toxicity of each synthetic compound was in silico assayed using three-dimensional quantitative structure-activity relationship (3D-QSAR) methods [26] in Derek^TM^ Nexus system (Lhasa Limited; Leeds, UK) [27]. To construct 3D-QSAR models, structural alignment and comparative molecular field analysis (CoMFA) were performed on the synthetic compounds for their predictive effectiveness in tyrosinase inhibition. The compounds were dissolved in dimethyl sulfoxide (DMSO; Sigma-Aldrich, St. Louis, MO, USA) at 100 mM concentration and freshly diluted in culture media.

### 4.2. Cell Culture

Mouse melanoma cell line, B16F10, was obtained from the Korean Cell Line Bank (Seoul, Korea). The cells were cultured in Dulbecco’s modified Eagle’s medium (DMEM) supplemented with 10% fetal bovine serum (FBS), penicillin (100 IU/mL), and streptomycin (100 mg/mL) (all from Invitrogen, Carlsbad, CA, USA). For sub-cultivation, cells were rinsed in phosphate-buffered saline (PBS; Gibco/Life Technologies, Carlsbad, CA, USA) and detached using 0.05% trypsin-ethylenediaminetetraacetic acid (EDTA; Gibco/Life Technologies). The cells were harvested, plated onto prepared culture dishes, and maintained in a humidified incubator (37 °C, 5% CO_2_).

### 4.3. Determination of Cytotoxicity

To test the cytotoxicity of each extract, cell counting kit-8 (CCK-8; Dojindo Laboratories, Kumamoto, Japan) was used, as previously described [28]. B16F10 cells were plated at a density of 5 × 10^3^ cells/well in a 96-well plate (Nunc™; Thermo Fisher Scientific, Waltham, MA, USA) and incubated with various concentrations of each compound for 24 h or 72 h. CCK-8 assay was performed as per manufacturer’s instructions. The absorbance, which is proportional to the number of living cells in each well, was measured at 450 nm wavelength using a microplate reader (Sunrise™, Tecan Group Ltd., Männedorf, Switzerland).

### 4.4. Determination of Relative Melanin Contents

B16F10 cells were plated at a density of 2 × 10^6^ cells in a 100 mm culture dish (Nunc™; Thermo Fisher Scientific) and treated with the compound at 1 or 2 μM and/or alpha-melanocyte-stimulating hormone (α-MSH) for 72 h. The cells were harvested after rinsing with PBS and the cell pellet was placed in 1 N sodium hydroxide (NaOH) for 20 min at 80 °C to solubilize melanin. The melanin content was determined by measuring the absorbance of the supernatant at 490 nm wavelength. The protein content in the supernatant was determined by Bradford assay using bovine serum albumin (BSA) as the protein standard [29]. The melanin content was adjusted by the amount of protein in the same reaction. The results were expressed as the percentage of the control.

### 4.5. Measurement of Tyrosinase Inhibitory Activity

In vitro tyrosinase activity was examined by measuring the rate of oxidation of l-DOPA (Sigma-Aldrich) [30] with minor modifications. In 840 μL of 100 mM phosphate buffer (pH 7.0) in the absence or presence of 50 μL of sample diluents, 50 μL of mushroom tyrosinase (2000 U/mL; Sigma-Aldrich) or B16F10 cell homogenates (3 mg/mL) was added and incubated at 37 °C for 5 min. Subsequently, 60 μL of 10 mM l-DOPA was added to yield a final concentration of 0.6 mM. The mixture was then allowed to react for 15 min (with mushroom tyrosinase) or 60 min (with cell homogenates). The reaction was monitored at 475 nm wavelength. A control reaction was performed with DMSO. The percentage of inhibition of tyrosinase activity was calculated as inhibition (%) = (A − B)/A × 100, where A represents the difference in the absorbance of the control between the incubation time of 5 and 20 min and B represents the difference in the absorbance of the test sample between the incubation time of 5 and 20 min. Resveratrol (10 μM) was used as a positive standard [31].

### 4.6. Quantitative Polymerase Chain Reaction (qPCR) Analysis

Total RNA extracts were prepared from the harvested cells using a column-based isolation kit (RNeasy Mini Kit; Qiagen, Hilden, Germany) according to the manufacturer’s instructions [32]. After quantification at 260 nm wavelength, RNA (0.5 μg) was reverse-transcribed to cDNA using Moloney murine leukemia virus (M-MLV) reverse transcriptase (Thermo Fisher Scientific) with oligo(dT)_12–18_ primer. To analyze the relative mRNA levels for each gene, SYBR Green-based real-time PCR was performed using LightCycler^®^ Multiplex Masters (Roche, Basel, Switzerland) with the designated primer sets (Table 1) on LightCycler^®^ Nano Instrument (Roche). The transcript expression levels were normalized with the expression level of β-actin.

### 4.7. Western Blot Analysis

Cultured cells were collected, rinsed, and subjected to NE-PER^®^ Nuclear and Cytoplasmic Extraction Reagents (Thermo Fisher Scientific) as previously described [32,33]. After protein quantification by Bradford assay, proteins were separated by 12% sodium dodecyl sulfate polyacrylamide gel electrophoresis (SDS-PAGE) and transferred onto a polyvinylidene fluoride (PVDF) membrane. Primary antibodies used in this study were as follows: rabbit anti-microphthalmia-associated transcription factor (MITF; Abcam, Cambridge, UK), goat anti-lamin B, mouse anti-tyrosinase (TYR), mouse anti-tyrosinase-related protein 1 (TYRP1), mouse anti-TYRP2, and mouse anti-β-actin (Santa Cruz Biotechnology, Inc., Dallas, TX, USA). Secondary antibodies used were anti-mouse, anti-rabbit, or anti-goat IgG conjugated to horseradish peroxidase (Santa Cruz Biotechnology, Inc.). Protein bands were visualized using the SuperSignal^®^ West Pico Chemiluminescent Substrate kit (Thermo Fisher Scientific) and ImageQuant LAS 4000 mini (GE Healthcare Life Sciences, Little Chalfont, UK). Digital images were analyzed for densitometry using Image Studio^TM^ Lite software (LI-COR Corp., Lincoln, NE, USA).

### 4.8. Determination of Depigmentation in Zebrafish

The study was conducted in accordance with the guidelines of the Institutional Animal Care and Use Committee of the Korea Institute of Toxicology, Daejeon, Korea (permission number: 1604-0112; approved on 2 April 2017). Wild-type adult zebrafish (6–24-month-old) were cultured at the Systems Toxicology Research Center, Korea Institute of Toxicology. These were maintained under standard conditions (carbon-filtered dechlorinated tap water at 27 ± 1 °C with 14/10 h light/dark cycle), as previously described [34,35]. To test the anti-melanogenic effect of the selected compounds, the embryos were collected and exposed to the compounds at designated concentrations (0, 125, 250, 500, and 1000 μg/L). After 72 h of incubation, pigment development in zebrafish was observed under a microscope (Leica M205 FA, Leica, Wetzlar, Germany). Arbutin was used as a positive standard at concentrations of 0, 125, 250, 500, and 1000 mg/L. The obtained images of pigment area density were quantitatively analyzed using ImageJ software, 1.48v (developed at the National Institutes of Health), and normalized to those of the control

### 4.9. Statistical Analysis

The obtained data were analyzed by one-way analysis of variance (ANOVA) using SPSS statistics 22 software (SPSS Inc., Chicago, IL, USA). Statistical differences among experimental groups were determined by one-way ANOVA, followed by Duncan’s multiple range test at 5% significance level. Statistical difference (*p* < 0.05) among values was marked with different alphabetical letters.

## Figures and Tables

**Table 1:** Primer sets for real-time PCR.

Gene (NCBI Accession No.)	Primer (5′→3′)		Product Length (bp)
	Forward	Reverse	
*Tyr* (NM_011661)	CCTCCTGGCAGATCATTTGT	GGCAAATCCTTCCAGTGTGT	236
*Tyrp1* (NM_001282015)	CTTGGAGGTCCGTGTATTTG	GACCGCATCAGTGAAAGTGT	223
*Tyrp2* (NM_010024)	TACCATCTGTTGTGGCTGGA	CAAGCTGTCGCACACAATCT	204
*Mitf* (NM_001178049)	AGGACCTTGAAAACCGACAG	GTGGATGGGATAAGGGAAAG	115
*β-actin* (NM_007393)	ACTATTGGCAACGAGCGGTT	ATGGATGCCACAGGATTCCA	81

*Tyr*, tyrosinase; *Tyrp1*, tyrosinase-related protein 1; *Tyrp2*, tyrosinase-related protein 1; *Mitf*, microphthalmia-associated transcription factor.

## Supplementary Materials

Supplementary materials can be found at http://www.mdpi.com/1422-0067/19/4/1067/s1.
