# Supplementary material for: Novel (1E,3E,5E)-1,6-bis(Substituted phenyl)hexa-1,3,5-triene Analogs Inhibit Melanogenesis in B16F10 Cells and Zebrafish
Source: Int J Mol Sci. 2018 Apr 3;19(4):1067. doi: 10.3390/ijms19041067 (PMC5979499; doi:10.3390/ijms19041067)

Supplementary Table S1. Synthetic details of the tested compounds

Compound #	Chemical Name	Synthetic Details
1	(1*E*,3*E*,5*E*)-1,6-Diphenylhexa-1,3,5-triene	^{1}H NMR (500 MHz, DMSO-d_6) δ 7.54–7.48 (m, 4H), 7.35 (t, *J* = 7.6 Hz, 4H), 7.25 (t, *J* = 7.4 Hz, 2H), 7.06 (ddd, *J* = 15.6, 7.1, 3.2 Hz, 2H), 6.68 (d, *J* = 15.6 Hz, 2H), 6.62 (dd, *J* = 7.1, 3.0 Hz, 2H); MS (EI) *m/e* 231.9 (100%)[M+]
2	(1*E*,3*E*,5*E*)-1,6-Bis(2-methoxyphenyl)hexa-1,3,5-triene	^{1}H-NMR (300 MHz, $CDCl_3$) δ 7.54−7.48 (m, 2H), 7.26−7.21 (m, 2H), 7.02−6.92 (m, 6H), 6.99−6.93 (m, 2H), 6.59−6.55(m, 2H), 3.90 (s, 3H); MS (EI) *m/e* 292.9 [M+]
3	(1*E*,3*E*,5*E*)-1,6-Bis(2-(trifluoromethyl)phenyl)hexa-1,3,5-triene	^{1}H NMR (300 MHz, $CDCl_3$) δ 7.72 (d, *J* = 8.0 Hz, 2H), 7.64 (d, *J* = 7.8 Hz, 2H), 7.51 (t, *J* = 7.7 Hz, 2H), 7.32 (t, *J* = 7.8 Hz, 2H), 7.05–6.83 (m, 4H), 6.65–6.58 (m, 2H); MS (EI) *m/e* 368.8 (30%) [M+1]
4	(1*E*,3*E*,5*E*)-1,6-Bis(2-methoxyphenyl)hexa-1,3,5-triene	^{1}H-NMR (300 MHz, $CDCl_3$) δ 7.21−7.17 (m, 2H), 7.11−6.97 (m, 3H), 7.04−6.81 (m, 5H), 6.64−6.57 (m, 2H), 3.93 (s, 6H), 3.89 (s, 6H); MS (EI) *m/e* 353.0 [M+]
5	(1*E*,3*E*,5*E*)-1,6-Bis(4-ethoxyphenyl)hexa-1,3,5-triene	^{1}H NMR (500 MHz, $CDCl_3$) δ 7.37 (d, *J* = 7.9 Hz, 3H), 7.28 (t, *J* = 8.3 Hz, 4H), 6.88 (dd, *J* = 5.0 Hz, 3H), 6.52 (d, *J* = 8.2 Hz, 1H), 6.48–6.46 (m, 2H), 4.08–4.06 (m, 4H), 1.45–1.42 (t, *J*=7.0 Hz, 6H); MS (EI) *m/e* 320.0(100%) [M+]
6	4,4'-((1*E*,3*E*,5*E*)-Hexa-1,3,5-triene-1,6-diyl)bis(2-methoxyphenol)	^{1}H-NMR (500 MHz, $CDCl_3$) δ 6.98−6.92 (m, 4H), 6.95−6.87 (m, 2H), 6.79−6.72 (m, 2H), 6.55−6.47 (m, 4H), 5.66 (s, 2H), 3.96 (s, 6H); MS (EI) *m/e* 324.0 [M+]
7	(1*E*,3*E*,5*E*)-1,6-Di-p-tolylhexa-1,3,5-triene	^{1}H NMR (500 MHz, $CDCl_3$) δ 7.34 (d, *J* = 7.8 Hz, 4H), 7.16 (d, *J* = 7.8 Hz, 4H), 6.91–6.83 (m, 2H), 6.59 (d, *J* = 15.4 Hz, 2H), 6.51 (dd, *J* = 7.0, 2.9 Hz, 2H), 2.37 (s, 6H); MS (EI) *m/e* 260.1(100%) [M+]
8	(1*E*,3*E*,5*E*)-1,6-Bis(4-bromophenyl)hexa-1,3,5-triene	^{1}H NMR (500 MHz, $CDCl_3$) δ 7.51 (d, *J* = 8.3 Hz, 2H), 7.49–7.43 (m, 3H), 7.36 (d, *J* = 8.5 Hz, 1H), 7.27–7.21 (m, 2H), 6.86 (dd, *J* = 15.2, 11.2 Hz, 2H), 6.62–6.50 (m, 3H), 6.46–6.34 (m, 1H); MS (EI) *m/e* 391.3(28%)2Br [M+3]

9	(1*E*,3*E*,5*E*)-1,6-Bis(4-chlorophenyl)hexa-1,3,5-triene	^{1}H NMR (500 MHz, $CDCl_3$) δ<br>7.36 (td, *J* = 6.6, 3.3 Hz, 5H), 7.31 (dt, *J* = 6.3, 2.0 Hz, 4H), 6.87 (ddd, *J* = 15.5, 7.0, 3.0 Hz, 2H), 6.60–6.55 (m, 2H), 6.53 (dd, *J* = 7.0, 3.0 Hz, 1H); MS (EI) *m/e* 300.8(15%) [M+1]
10	(1*E*,3*E*,5*E*)-1,6-Bis(2-phenoxyphenyl)hexa-1,3,5-triene	^{1}H NMR (300 MHz, $CDCl_3$) δ<br>7.39–7.26 (m, 6H), 7.17–6.99 (m, 10H), 6.90–6.77 (m, 4H), 6.58–6.45 (m, 4H); MS (EI) *m/e* 417.1(28%) [M+1]
11	(1*E*,3*E*,5*E*)-1,6-Dimesitylhexa-1,3,5-triene	^{1}H NMR (300 MHz, $CDCl_3$) δ<br>6.88 (s, 4H), 6.61 (d, *J* = 15.0 Hz, 2H), 6.50–6.34 (m, 4H), 2.29 (d, *J* = 11.3 Hz, 18H); MS (EI) *m/e* 317.0(20%) [M+1]
12	(1*E*,3*E*,5*E*)-1,6-Bis(4-fluoro-3-methoxyphenyl)hexa-1,3,5-triene	^{1}H NMR (300 MHz, $CDCl_3$) δ<br>7.09–7.00 (m, 4H), 6.95 (ddd, *J* = 8.5, 4.6, 2.0 Hz, 2H), 6.80 (ddd, *J* = 15.4, 7.1, 3.1 Hz, 2H), 6.59–6.49 (m, 3H), 3.95 (s, 6H); MS (EI) *m/e* 328.2(70%) [M+1]
13	(1*E*,3*E*,5*E*)-1,6-Bis(2,5-dibromophenyl)hexa-1,3,5-triene	^{1}H NMR (300 MHz, $CDCl_3$) δ<br>7.70 (dd, *J* = 4.5, 2.4 Hz, 1H), 7.54–7.37 (m, 3H), 7.29 (d, *J* = 2.4 Hz, 1H), 7.19 (ddd, *J* = 8.5, 4.3, 2.3 Hz, 1H), 6.96–6.75 (m, 2H), 6.70–6.61 (m, 2H), 6.50–6.34 (m, 2H); MS (EI) *m/e* 541.2(4 Br) [M-1]
14	(1*E*,3*E*,5*E*)-1,6-Bis(5-fluoro-2-methoxyphenyl)hexa-1,3,5-	^{1}H NMR (300 MHz, $CDCl_3$) δ<br>7.23–7.08 (m, 2H), 7.00–6.74 (m, 8H), 6.59–6.51 (m, 2H), 3.83 (d, *J* = 7.6 Hz, 6H); MS (EI) *m/e* 328.9(100%) [M+1]
15	(1*E*,3*E*,5*E*)-1,6-Bis(2,4-dichlorophenyl)hexa-1,3,5-triene	^{1}H NMR (300 MHz, $CDCl_3$) δ<br>7.57–7.29 (m, 4H), 7.21 (dd, *J* = 9.0, 2.4 Hz, 2H), 6.93 (d, *J* = 3.2 Hz, 3H), 6.69–6.45 (m, 3H); MS (EI) *m/e* 369.9(40%) (4 Cl)[M+2]
16	(1*E*,3*E*,5*E*)-1,6-Bis(2-fluoro-5-methoxyphenyl)hexa-1,3,5-triene	^{1}H NMR (300 MHz, $CDCl_3$) δ<br>7.02–6.87 (m, 6H), 6.77–6.68 (m, 4H), 6.56 (dd, *J* = 6.9, 3.0 Hz, 2H), 3.81 (s, 6H); MS (EI) *m/e* 329.1(100%) [M+1]
17	(1*E*,3*E*,5*E*)-1,6-Bis(3-(2-chlorophenyl)-1-phenyl-1*H*-pyrazol-4-yl)hexa-1,3,5-triene	^{1}H NMR (300 MHz, $CDCl_3$) δ<br>8.10 (s, 2H), 7.78–7.71 (m, 4H), 7.54–7.43 (m, 8H), 7.40–7.34 (m, 4H), 7.33–7.28 (m, 2H), 6.47 (ddd, *J* = 15.6, 7.0, 3.1 Hz, 2H), 6.30–6.18 (m, 4H); MS (EI) *m/e* 584.9(100%) [M+1]
18	(1*E*,3*E*,5*E*)-1,6-Bis(3-(3-((2,6-difluorobenzyl)oxy)-4-methoxyphenyl)-	^{1}H NMR (300 MHz, $CDCl_3$) δ

	1-phenyl-1*H*-pyrazol-4-yl)hexa-1,3,5-triene	8.13 (d, *J* = 10.5 Hz, 2H), 7.78 (d, *J* = 8.1 Hz, 4H), 7.50–7.43 (m, 6H), 7.33–7.28 (m, 6H), 7.02–6.87 (m, 8H), 6.56 (d, *J* = 15.5 Hz, 2H), 6.41 (dd, *J* = 6.7, 3.0 Hz, 2H), 5.25 (s, 4H), 3.89 (d, *J* = 6.3 Hz, 6H); MS (EI) *m/e* 860.9 (100%) [M+1]
19	(1*E*,3*E*,5*E*)-1,6-Bis(6-bromopyridin-2-yl)hexa-1,3,5-triene	^{1}H NMR (300 MHz, $CDCl_3$) δ 8.37 (d, *J* = 10.5 Hz, 2H), 7.60 (d, *J* = 8.1 Hz, 3H), 7.44–7.42 (d, 2H), 6.93–6.89 (m, 2H), 6.56 (d, *J* = 15.5 Hz, 3H); MS (EI) *m/e* 392.8(100%) (2Br)[M+3]
20	(1*E*,3*E*,5*E*)-1,6-Bis(3-(3,4-bis(difluoromethoxy)phenyl)-1-phenyl-1*H*-pyrazol-4-yl)hexa-1,3,5-triene	^{1}H NMR (300 MHz, $CDCl_3$) δ 8.10 (d, *J* = 16.0 Hz, 2H), 7.83–7.70 (m, 4H), 7.65 (s, 2H), 7.61–7.55 (m, 2H), 7.50 (q, *J* = 7.6 Hz, 4H), 7.34 (dd, *J* = 12.9, 7.5 Hz, 4H), 6.88–6.65 (m, 3H), 6.63–6.52 (m, 3H), 6.50 (s, 1H), 6.45–6.25 (m, 3H); MS (EI) *m/e* 780.9(100%) [M+1]
21	(1*E*,3*E*,5*E*)-1,6-Bis(3-(2-bromophenyl)-1-phenyl-1*H*-pyrazol-4-yl)hexa-1,3,5-triene	^{1}H NMR (300 MHz, $CDCl_3$) δ 8.17–8.07 (m, 2H), 7.83–7.68 (m, 6H), 7.55–7.39 (m, 9H), 7.36–7.27 (m, 4H), 6.52–6.35 (m, 2H), 6.32–6.16 (m, 3H); MS (EI) *m/e* 674.8(100%) (2Br)[M+2]
22	(1*E*,3*E*,5*E*)-1,6-Bis(2-chloro-6-fluorophenyl)hexa-1,3,5-triene	^{1}H NMR (300 MHz, $CDCl_3$) δ 7.24–6.94 (m, 8H), 6.80 (d, *J* = 15.8 Hz, 2H), 6.64–6.56 (m, 2H); MS (EI) *m/e* 335.5[M-1]
23	(1*E*,3*E*,5*E*)-1,6-Bis(3-(2-chloro-4-methoxyphenyl)-1-phenyl-1*H*-pyrazol-4-yl)hexa-1,3,5-triene	^{1}H NMR (300 MHz, $CDCl_3$) δ 8.11 (d, *J* = 5.2 Hz, 1H), 7.76 (d, *J* = 7.1 Hz, 4H), 7.57 (dd, *J* = 8.6, 2.0 Hz, 3H), 7.51–7.42 (m, 6H), 7.32 (d, *J* = 6.8 Hz, 3H), 7.03 (d, *J* = 8.3 Hz, 3H), 6.53 (d, *J* = 15.3 Hz, 2H), 6.42 (d, *J* = 12.7 Hz, 2H), 4.00 – 3.94 (m, 6H); MS (EI) *m/e* 644.91(100%)[M+1]
24	(1*E*,3*E*,5*E*)-1,6-Bis(5-methylthiophen-2-yl)hexa-1,3,5-triene	^{1}H NMR (300 MHz, $CDCl_3$) δ 6.75 (d, *J* = 3.5 Hz, 2H), 6.65–6.48 (m, 6H), 6.36 (dd, *J* = 6.4, 3.0 Hz, 2H), 2.46 (d, *J* = 1.1 Hz, 6H); MS (EI) *m/e* 271.9(100%) [M+]
25	(1*E*,3*E*,5*E*)-1,6-Bis(3,4,5-trimethoxyphenyl)hexa-1,3,5-triene	^{1}H NMR (300 MHz, $CDCl_3$) δ 6.80 (ddd, *J* = 15.3, 7.0, 3.0 Hz, 2H), 6.64 (s, 4H), 6.57–6.48 (m, 4H), 3.90 (s, 12H), 3.86 (s, 6H); MS (EI) *m/e* 412.8(100%) [M+1]

Supplementary Figure S1. A total of 25 synthetic compounds and their chemical identities.

Compound #1

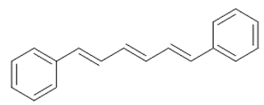

#2

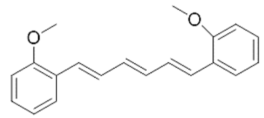

#3

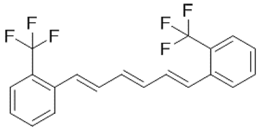

#4

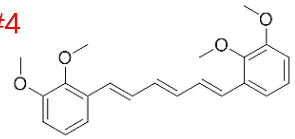

#5

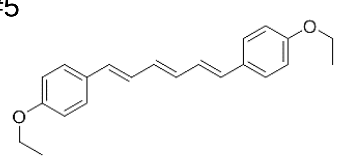

#6

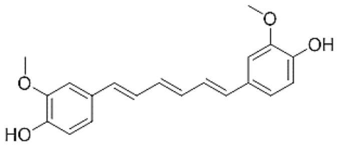

#7

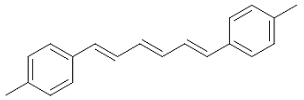

#8

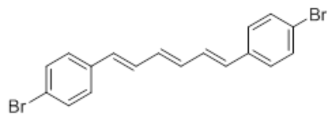

#9

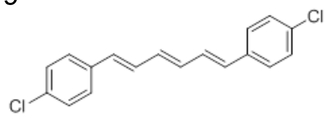

#10

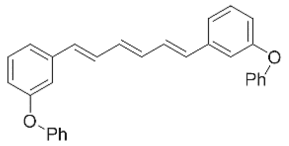

#11

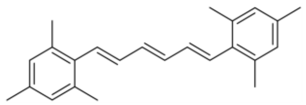

#12

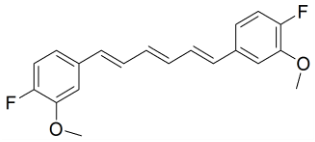

#13

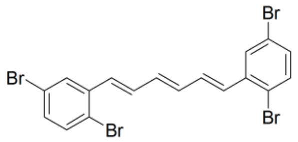

#14

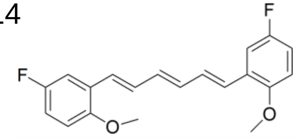

#15

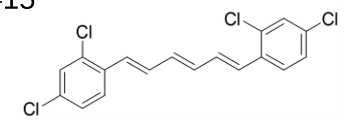

#16

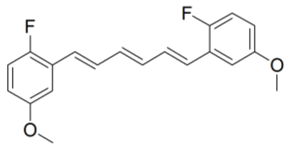

#17

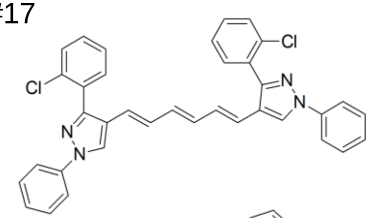

#18

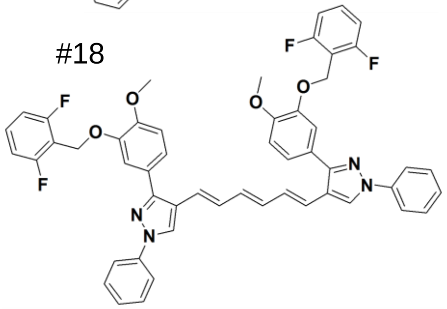

#19

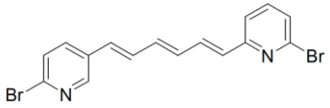

#20

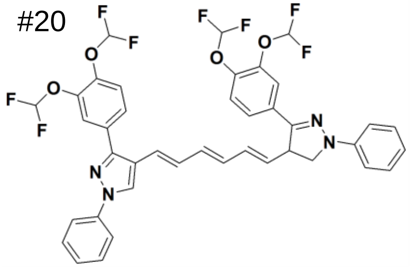

#21

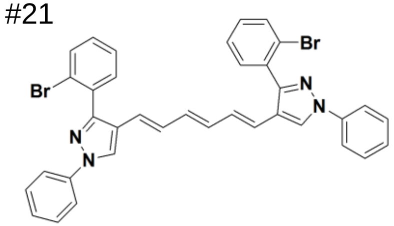

#22

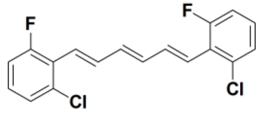

#23

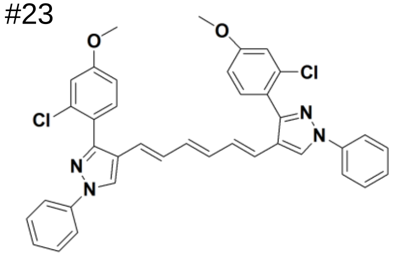

#24

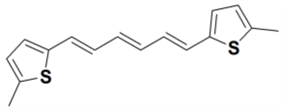

#25

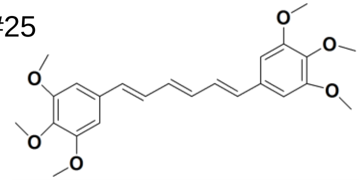

Supplementary Figure S2. Cytotoxicity of the synthetic compounds. B16F10 murine melanoma cells were treated with each compound at 0, 1, 2, and 5 μM for 72 h. CCK-8 assay was performed to assess the cytotoxic effect. N = 3; error bars, mean ± SD. Different alphabetical letters indicate significant differences among the conditions ($p < 0.05$).

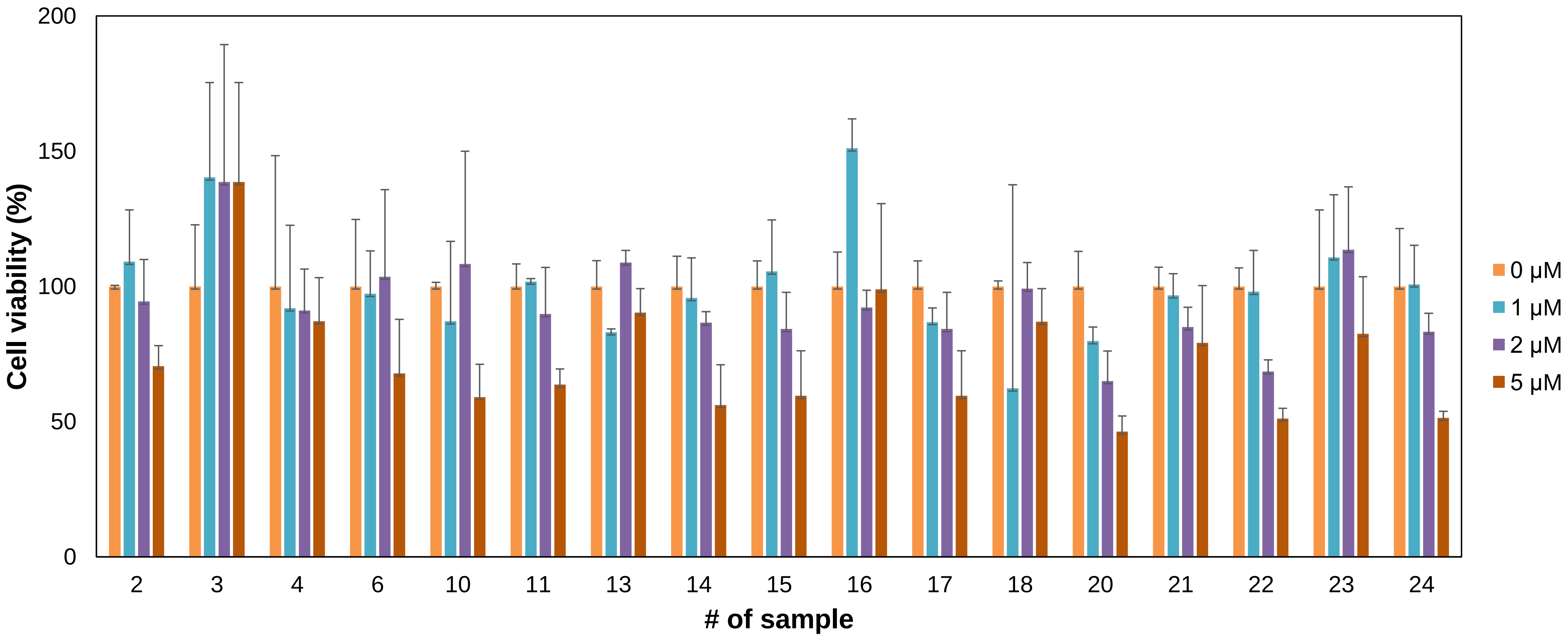

Supplementary Figure S3. Cellular tyrosinase effect of compound #2, #4, and #6. Catalytic activity of crude enzyme solution from B16F10 cells was measured in the presence of each compound at concentrations of 0, 6.25, 12.5, 25, and 50 μM. N = 3; error bars, mean ± SD. Different alphabetical letters indicate significant differences among the conditions ($p < 0.05$).

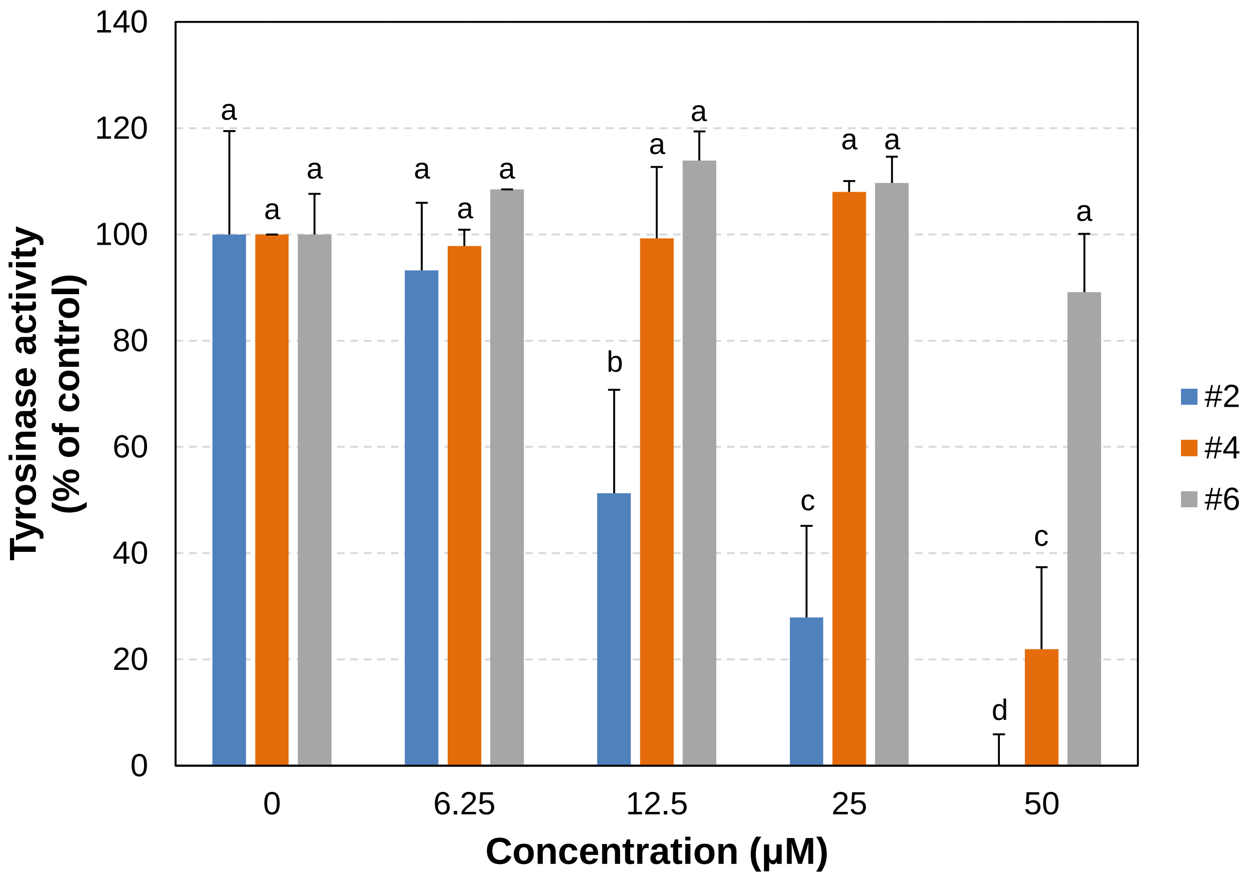

Supplement: Supplementary file 1 [file ijms-19-01067-s001.pdf]
